# The power of 4th industrial revolution in the fashion industry: what, why, and how has the industry changed?

**DOI:** 10.1186/s40691-021-00259-4

**Published:** 2021-10-25

**Authors:** Byoungho Ellie Jin, Daeun Chloe Shin

**Affiliations:** 1 Professor, Department of Textile and Apparel, Technology and Management, Wilson College of Textiles, North Carolina State University, 1020 Main Campus Drive, Raleigh, NC 27695 USA; 2 Graduate Student, Department of Textile and Apparel, Technology and Management, Wilson College of Textiles, North Carolina State University, 1020 Main Campus Drive, Raleigh, NC 27695 USA

**Keywords:** Fourth industrial revolution, Technology, Business model innovation, Personalization, Sustainability, Productivity

## Abstract

The 4th Industrial Revolution (4IR henceforth) is fundamentally reshaping the way we live and work. Each industrial revolution has evolved to solve major problems in society. This study views unmatched demand and oversupply as the major problems in the fashion industry and posits that 4IR technologies are being deployed to solve these problems by addressing three prime goals—hyper-personalization, environmental sustainability, and productivity. Based on a literature review and analyses of global industry cases, this study examines what, why, and how the 4IR technologies address these three prime goals. By comparing successful cases that do not utilize the 4IR technologies with those that do, this study highlights that innovative business models that address the unmet needs of the consumers are more important than technology adoption per se. Drawn from ample global cases, the findings can offer strategic directions for fashion firms preparing for unforeseeable changes that are further being accelerated by the Covid-19 pandemic. This study concludes with insights into how 4IR is shaping the fashion industry and raises thought-provoking questions for the industry and academia.

## Introduction

The 4th Industrial Revolution (4IR henceforth) is fundamentally reshaping the way we live and work. In each industrial revolution, inventions and new technologies contributed to the enhancement of industries and human lives through mechanization during the 1st Industrial Revolution, mass production and electricity during the 2nd, and IT systems and automation during the 3rd. As with the previous industrial revolutions, numerous technologies have enabled the 4th Industrial Revolution: robotics, intelligent manufacturing, augmented and virtual reality, and artificial intelligence, to name a few. The focus of every industrial revolution in history was never the development of technologies itself. Rather, it was about enhancing the quality of human life through enhanced productivity, which was made possible by inventions and innovative technologies. Then, what are the aims of the 4th Industrial Revolution in the context of the fashion industry, which includes a wide range of product categories, from apparel and footwear, jewelry, to bags and accessories? With the 4IR technologies, what is the fashion industry seeking to achieve?

Given that the ultimate goal of technology development is solving industry challenges to better serve human needs (Schwab, 2016), it is logical to first identify unique challenges in the fashion industry and then to discuss how technologies are being applied to address them. Yet, a comprehensive review of what, why, and how the 4IR technologies are being applied in the fashion industry is limited. Prior research has primarily focused on a single technology, whether it be robotics and intelligent manufacturing (Acaccia et al., 2003; Michelini & Razzoli, 2013), 3D printing (Perry, 2018; Sun & Zhao, 2017; Vanderploeg et al., 2017), virtual and augmented reality (Kim & Forsythe, 2008; Park et al., 2018; Shim & Lee, 2011), or artificial intelligence (Guo et al., 2011; Liang et al., 2019). While there are a few studies that offer a comprehensive overview of the technology’s implications for the fashion industry, they are still limited in scope (Bertola & Teunissen, 2018; Braglia et al., 2020). They mainly discuss benefits for companies as opposed to consumers, such as improving efficiency in the supply chain and staff training (Braglia et al., 2020), or lack a thorough discussion on the role of business models (Bertola & Teunissen, 2018), despite their central role in determining the commercial value of a technology (Chesbrough, 2010).

To better understand the role of the 4IR technologies in the global fashion industry, this study examines what, why, and how these technologies are being applied to address major challenges in the industry from a macro perspective. This study posits that these technologies are being deployed to solve three prime concerns of both the industry and consumers—productivity, environmental sustainability, and hyper-personalization. Further, this study posits that these concerns can also be addressed with non-technology based innovations, such as business model innovations.

This study begins with a brief overview of former industrial revolutions, focusing on their impact on the industry. Following an analysis of major challenges in the industry, three prime industry and consumer concerns (i.e., productivity, environmental sustainability, and hyper-personalization) are proposed to examine what, why, and how the 4IR technologies fulfill the concerns. Furthermore, this study identifies different types of fashion companies that fulfill the prime concerns without relying heavily on 4IR technologies. Those companies are largely DTC (direct to consumer) start-ups that sell to consumers directly via online without any intermediaries, such as wholesalers and retailers (Jin & Shin, 2020). Their success in fulfilling the prime concerns is fundamentally driven by business model innovations.

By contrasting the two types of successful cases (i.e., those that capitalize on the 4IR technologies and those whose success is driven by business model innovations), this study highlights that developing an innovative business model that addresses major industry concerns is more crucial than adopting an emerging technology per se. While advanced technologies play a critical role, as they allow companies to deliver new customer values at scale, they are not a prerequisite for market disruptions. This study concludes with insights into how 4IR is shaping the fashion industry and raises thought-provoking questions for the industry and academia.

## Literature review

### Brief overview and prime aim of industrial revolutions

Each industrial revolution radically transformed economic systems and social structures (Schwab, 2016). The fashion industry was no exception. Between 1760 and 1840, the 1st industrial revolution ushered in mechanical production, powered by water and steam. Between the late 1800s and early 1900s, the 2nd industrial revolution enabled mass production, powered by electricity and the assembly line. The prime goal of the first two industrial revolutions was to enhance production productivity through mechanization and automation. Following a series of inventions, such as the spinning jenny, spinning mule, and assembly line, fashion production has moved away from the traditional system of craft-based production (Duarte et al., 2018).

The 3rd Industrial Revolution, which occurred in the mid twentieth century, focused on maximizing already enhanced productivity, based on automation and optimization. It was enabled by computer aided design and manufacturing systems, which significantly increased production speed, flexibility, and precision (Duarte et al., 2018). Beyond the production side, the information and communication technologies gave rise to new business models, such as fast fashion and e-commerce (Abnett, 2016). As a result, companies such as Inditex, H&M, and Amazon emerged as top retailers. Furthermore, the hyper-connectivity and the rise of social media platforms, such as Facebook and Instagram, have transformed marketing and branding strategies (Gensler et al., 2013).

The 4th Industrial Revolution builds on the previous digital revolution and capitalizes on the synergistic effects of various advanced technologies (Philbeck & Davis, 2019). The technologies encompass wide-ranging fields, including artificial intelligence, robotics, the internet of things, 3D printing, virtual and augmented reality (Schwab, 2016). Underlying these technologies is the power of digitization and information technology. That is, all these technologies are enabled and enhanced by digital power. For example, advanced robots rely on artificial intelligence, which is powered by computing power. While digital technologies are not new, they are becoming more sophisticated (Schwab, 2016). The 4th Industrial Revolution is expected to reach virtually every industry from transportation and health to banking (Iansiti & Lakhani, 2020).

In the fashion industry, the first three industrial revolutions had evolved to solve the problem of inefficiency in the production system. During the 1st industrial revolution, technologies were invented and applied to increase production output. This effort to increase productivity continued through the 2nd Industrial Revolution, culminating in factories of mass production (Allen, 2011). During the 3rd Industrial Revolution, to beat the heightened competition in the global economy brought on by the internet, new technologies were deployed to maximize productivity, further reducing the additional cost of producing one more unit of a good or service (Liu & Grusky, 2013). The primary value delivered by the first three industrial revolution technologies was providing sufficient products and services at a reduced price and time through enhanced productivity. Then what will be the major goals of the 4th Industrial Revolution? Are we continuously heading toward further enhancing productivity, such as increasing production volume and offering products at a lower price? What changes are the 4IR technologies bringing to the industry? To answer this question, it is important to identify the most pressing needs of industry and consumers because each Industrial Revolution has evolved to solve the major concerns in society.

### Three prime goals of the 4th industrial revolution to solve major concerns in the fashion industry

This study posits that the 4th industrial revolution has evolved to address the most pressing needs of industries and consumers. We identified two major concerns in the fashion industry and the 4IR technologies being utilized to solve these major issues. Many challenges in the fashion industry are deemed to result from one phenomenon: unmatched demand. Almost every fashion firm encountered this issue because it stems from the current business model that most apparel companies employ: a push supply chain system (Christopher et al., 2004; Fisher, 1997; Jin & Shin, 2020). In this current approach, companies produce goods based on forecasted demand and market the products to sell. However, unlike other product categories, the demand for apparel goods is hard to predict (Jin, 2004, 2006; Jin et al., 2011). The push supply approach inevitably results in a huge discrepancy between the forecasted and actual demand. After a season, therefore, this results in heavy markdowns and excess inventory that erode profits. A study by Bain & Co. estimated that the industry average markdown ratio is approximately 50% (Sull & Turconi, 2008). Such traditional business model is clearly unsustainable.

Traditional apparel companies that operate on the push supply chain have been struggling well before the Covid-19 pandemic broke out. The negative impacts of the pandemic are so severe that some incumbent apparel brands and retailers may no longer exist in the near future. Leading retailers from high-end (e.g., Nordstrom, Neiman Marcus) to low-end (e.g., JC Penny) and well-known brands such as Brooks Brothers, J Crew, and Victoria Secret have recently announced filing for Chapter 11 bankruptcy protection (Aleksander, 2020).

The next problem pointed out by many industry thought leaders is oversupply, supplying much more than consumers can consume (Dart & Lewis, 2017). Supply has skyrocketed, thanks to enhanced productivity enabled by the inventions and technologies from the former industry revolutions as well as global sourcing in low-cost manufacturing countries. In contrast, demand has plummeted in Western countries. Factors that contributed to the demand shrinkage include a growth of aging populations, and an emphasis on experiences and shared goods, rather than ownership (Dart & Lewis, 2017; Eventbrite, 2014; Goldman et al., 2017).

Such an irreconcilable gap between the supply and demand, owing to unmatched demand and oversupply, invariably leads the industry unsustainable to the environment. Apparel industry is one of the top polluting industries, responsible for 10% of humanity’s carbon emissions; more emissions than all international flights and maritime shipping combined (UNEP, 2018). Recent popular concepts of fast fashion have facilitated the idea of disposable clothes, which is an added concern to the industry’s environmental impact. This has led to a heightened awareness of environmental sustainability among customers, which has pushed fashion companies to direct their efforts towards sustainable production. Environmental sustainability is indeed one of the key trends and performance goals of most industries. Therefore, this study posits that one of the goals of 4th Industrial Revolution is solving consumer and industry concern toward environmental sustainability.

The next concern raised from unmatched demand and oversupply is the industry’s inability to meet consumer demand accurately. This is quite ironic given that the former industrial revolutions and global sourcing practice has made it possible to produce a vast amount of clothes at a reasonable price. Due to the forecast-driven push supply chain in the fashion industry, an average error in the forecast at the time of production is 40–100%, which results in an average stock-out rate of 10–40%, as compared to 1–2% for functional goods (Fisher, 1997).

To handle this inefficiency, the concept of mass customization was applied to meet the consumers’ demand more accurately (Senanayake & Little, 2010). In mass customization, fashion companies modularize a product by offering a range of options for different components of the product. For example, NikeID allows customers to customize shoes by choosing a combination of options for the outsole, midsole, upper, and the Swoosh. Similarly, Levi’s gives their customers the option to customize their jeans by choosing an individualized combination of the wash, overdye, pattern, distress, and back patch. Size and fit can be further customized based on the measurements taken with 3D body scanners (Lang et al., in press) or reported by the customers (Son of a Tailor, n.d.). Further examples include Sumissura, a women’s tailored clothing brand, and Knot Standard, a men’s custom clothing brand. The above examples, however, have achieved limited success in meeting consumer demand accurately to the exact sizes, fits, tastes, and preferences mainly because of the lack of scalable technologies (Paul, 2001). This study posits that the 4IR technologies will be utilized to meet consumer demand accurately by providing hyper-personalized products and services on a large scale.

The third prime goal of 4th Industrial Revolution is posited to be productivity because solving the problems of unmatched demand and oversupply requires efficient and effective solutions to the costly methods of individual demand forecasting and customized production (Paul, 2001). Here, we define productivity broadly because the traditional concept of productivity—increasing production volume at lower prices—may be incompatible with the two prime goals of environmental sustainability and personalization. As such, fashion companies should enhance demand accuracy to meet their personalization goal and produce the right amount to meet their environmental sustainability goal. Therefore, the productivity goal of the 4th Industrial Revolution should be differently conceptualized from the ones in the previous eras. This is vital as productivity, after all, helps achieve goals and is not separate from them.

Each industrial revolution has evolved to solve major problems in the industry. Based on the reasoning above, this study posits that the prime goals of the 4th Industrial Revolution include environmental sustainability, hyper-personalization, and productivity. In other words, the 4IR has evolved to address the three primary concerns stemming from the chronic issues of the fashion industry, that is, accurately meeting consumer needs sustainably and productively.

These three prime goals also manifest in statements by leading thought groups and their reports. The three goals are among the five key performance indicators of the World Economic Forum’s global manufacturing community, which include productivity, sustainability, customization, agility, and speed to market. The global manufacturing community are leaders in using 4IR technologies to innovate factories, value chains, and business models to address the diverse needs of various stakeholders along the value chains (World Economic Forum, 2020). As the manufacturing community strives to fulfil the most compelling needs of diverse stakeholders, including consumers and retailers, their performance indicators are deemed to represent the prime goals they aim to achieve. We view the last two goals of agility and speed to market as part of productivity, which has been broadly defined in this study. In the following, we further detail our definition of each prime goals in relation to the fashion industry. We conceptualize each goal encompassing both products and services and both industry and consumers. The research stream in each of prime goals are also analyzed briefly.

### Definition and literature on three prime goals

#### Environmental sustainability

In this study, among the three dimensions of sustainability, we focus on environmental sustainability, concerned with protecting and preserving ecological health, because environmental harm is a major consequence of unmatched demand and oversupply (Elkington, 1998). We examine environmental sustainability from the perspectives of the major stakeholders in the supply chain: manufacturers, retailers, and consumers.

The previous era’s focus on enhancing productivity increased supply at lower prices, which in turn fueled consumption (Matsuyama, 2002). Fast fashion, popularized since the early 2000s, has pushed over-consumption and disposability to the mainstream with its successful business model: offering a large variety of trendy products at cheap prices (Jung & Jin, 2016).

Recently, however, increasing consumer awareness of the negative impacts of mass producing garments, such as the depletion of natural resources and the use of toxic chemicals, has led to a push for environmentally sustainable development and consumption (Todeschini et al., 2017). This shift in consumer behavior is well represented in a recent global survey where 73% of the respondents said that they would definitely change their consumption habits to reduce their environmental impact (Nielsen, 2018). Likewise, fashion business executives globally have reported that environmental sustainability is the single biggest challenge for the industry in 2020 (Business of Fashion, 2020).

There is a large body of research on environmental sustainability in the fashion industry from multiple perspectives and on a range of topics, including consumer attitude toward sustainable materials (e.g., organic cotton; Hustvedt & Dickson, 2009) and product labeling (e.g., Atkinson & Rosenthal, 2014), the determinants of consumers’ sustainable apparel purchase behavior (e.g., Dhir et al., 2021), as well as sustainable business models (Stål & Corvellec, 2018; Thorisdottir & Johannsdottir, 2019) and manufacturing (Islam et al., 2020). Recently, more researchers are paying attention to emerging methods for achieving environmental sustainability, such as blockchain technology (e.g., Bullón Pérez et al., 2020).

#### Hyper-personalization

Our definition of hyper-personalization is inclusive of both customization and personalization, thus offering solutions tailored to consumers’ specific needs, be it tangible, such as a product, or intangible, such as variety. These needs can be achieved through various means, from offering a marketing mix precisely tailored to an individual based on big data (i.e., personalization; Jain et al., 2021), and allowing the customer to specify one or more elements of the marketing mix through modularization (i.e., customization; Arora et al., 2008), to offering access as opposed to ownership to a wide range of assortments and variants for the customer to choose from (i.e., rental model; Mukendi & Henninger, 2020). Our definition of hyper-personalization encompasses all these different methods, both conventional and emerging, that are used to achieve the common goal of satisfying specific needs of an individual customer. We will examine hyper-personalization for both products and services from multiple perspectives, including manufacturers, retailers, and consumers. In the fashion industry, a gap still exists between customer demand for hyper-personalization in products and shopping experiences (Epsilon Marketing, 2018). Cognizant of this unmet need, the industry is striving to bridge this gap. According to a recent survey of 200 marketing leaders by Forbes Insights, 57% of respondents reported that they seek to augment personalization capability in the next year (Forbes, 2019).

In academia, there is a significant body of research focused on understanding the benefits of hyper-personalization across the marketing mix, such as advertising (e.g., van Doorn & Hoekstra, 2013), product (Moon et al., 2008), and customer shopping experience (Häubl & Trifts, 2000). Prior research on product customization has focused on identifying the antecedents of consumer purchase intention, such as brand characteristics (e.g., Cho & Fiorito, 2009) and consumer characteristics (e.g., Chen-Yu & Yang, 2020; Ulrich et al., 2003), as well as consumer motivations (e.g., Fiore et al., 2004), the determinants of consumer satisfaction with customized products (e.g., Yoo & Park, 2016), and profiling customization demands (e.g., Lee et al., 2002; Pallant et al., 2020). The technologies for customization, such as CAD system, (Satama et al., 2011) and effective marketing strategy (e.g., Franke et al., 2009) have also been researched. There is a nascent stream of research on consumer adoption intention toward personalization enabled by 4IR technologies, such as big data analytics (e.g., Jain et al., 2021) and augmented reality (e.g., Smink et al., 2020). However, recent research is more focused on services, such as a curated styling service, enabled by big data and artificial intelligence (Sebald & Jacob, 2018; Tao & Xu, 2018; Woo & Ramkumar, 2018).

#### Productivity

Finally, productivity is defined as an effective and efficient transformation of input resources into output (Grönroos & Ojasalo, 2004), where the input resources are time, efforts, and money (Johnston & Jones, 2004). As production productivity was largely achieved by prior industrial revolutions, productivity during the 4IR focuses on decision-making processes, beyond the traditional definition of producing more products at lower costs (Vaidya et al., 2018). It pertains to enhancing efficiency—“doing things right”—and effectiveness—“doing the right things”—not only in production, but also in consumer and company decision-making processes (Anitsal & Schumann, 2007). Therefore, factors such as accuracy, convenience, and speed in decision-making are now as important as accuracy and efficiency in production.

As with any industry, productivity underlies any business activity in the fashion industry, particularly in accelerating and scaling up production and process automation (PwC, 2020). Hyper-personalization calls for agile on-demand production in smaller batch sizes and shorter lead times (Paul, 2001). To scale up hyper-personalization, what needs to accompany is enhanced accuracy and efficiency in the process of forecasting demand for an individual customer (Paul, 2001). Thus, handling and analyzing enormous amounts of data, both at the aggregate and individual level, is an extremely resource-intensive task if it is not automated (Koehler, 2018).

From the consumer perspective, productivity in terms of convenience is receiving greater attention. Consumers are increasingly demanding that their shopping experiences require less time, effort, and money. For example, 83% of U.S. consumers report that convenience while shopping is more important now when compared to 5 years ago (NRF, 2020). Following groceries, clothing is ranked the second product category that consumers are willing to pay more for to increase convenience (NRF, 2020). Along with fast delivery, easy checkout, and hassle-free returns, consumers want to expend less effort and time finding the right product and the right size. These needs are even more important to address as digital channels have become the primary means for shopping (Briedis et al., 2020). Although the retailers are making efforts to provide digital solutions, such as size recommendation systems, a majority of consumers report that they want more convenience in their online shopping experience (NRF, 2020).

In academia, from the manufacturing perspective, researchers have examined factory productivity levels (Bheda et al., 2003), the use of technologies, such as robotics (Michelini & Razzoli, 2013) and radio frequency identification (Nayak et al., 2015), and manufacturers’ technology adoption (Varukolu & Park‐Poaps, 2009). Prior research on consumer and retailing has examined the role of convenience across the consumer shopping journey, including product search (e.g., Shim et al., 2001), payment (e.g., de Kerviler et al., 2016), and multichannel experience (e.g., Dholakia et al., 2010). In recent years, there is a growing interest in enhancing productivity via 4IR technologies, such as augmented reality and artificial intelligence, both in manufacturing (e.g., Braglia et al., 2020) and the consumer shopping experience (e.g., Grewal et al., 2020). The next section illustrates *what* the 4IR technologies are and *how* they are deployed to address the three prime goals with company cases.

## Methods

This study took an integrative literature review approach by integrating the literature on technology-based and non-technology based innovations and analyzing diverse company cases. This approach is appropriate for research on an emerging topic that would benefit from a holistic conceptualization and integration of the literature (Snyder, 2019). The goal is to synthesize the literature to offer a new perspective (Torraco, 2005). Therefore, when selecting company cases, we employed purposive sampling, as it offers the most representative cases. Purposive sampling is a typical selection method in case studies where cases that possess the characteristics of interest are selected as they offer the most pertinent and rich information (Etikan et al., 2016). The sampling criteria was whether the company deployed either at least one of the 4IR technologies or a business model innovation. To identify the representative cases, we conducted a broad web search using a combination of these keywords: robotics, intelligent manufacturing, 3D printing, 3D knitting, virtual and augmented reality, artificial intelligence, big data, business model, innovation, and startup. They were combined with at least one of the following terms: fashion, garment, and apparel. The sources of data obtained include academic journals and conference proceedings as well as trade articles and books.

## Results

### Meeting the prime goals utilizing 4IR technologies

This section gives an overview of the application of 4IR technologies relevant to the fashion industry: robotics and intelligent manufacturing, 3D printing and knitting, virtual and augmented reality, and artificial intelligence. What follows is a discussion of *how* these technologies address the three prime goals identified above.

#### Robotics and intelligent manufacturing

Coupled with other enabling technologies, such as artificial intelligence, sensor technologies, and computing power, robotics’ application extends beyond simple high-volume production activities. Powered by these advanced technologies, intelligent manufacturing, also known as the smart factory, pushes the boundaries of traditional automation even further. As a fully connected and flexible system, a smart factory can self-optimize by adapting to changing demands in real-time and run the production processes with minimal human intervention and high reliability (Radziwon et al. 2014). It can do so by collecting and analyzing real-time data from connected production systems as well as historical data (Wang et al., 2016). This data-driven manufacturing is more efficient and agile, resulting in less production downtime. Its ability to predict and fix problems and to adjust to changes in the facility lead to a competitive advantage (Zhong et al., 2017).

In the fashion industry, the advancement in robotics is beginning to automate a traditionally labor-intensive task: sewing. With machine vision, sewing robots can detect distortions in fabrics and make necessary adjustments (Emont, 2018). With fully automated T-shirt production lines, a sewing robot at Tianyuan Garments, a major producer of sportswear brands such as Adidas and Reebok, can cut fabrics and sew a T-shirt in about four minutes. With 21 fully operational production lines, automation is expected to cut manual labor by 90%, lowering the cost for each T-shirt to 33 cents (Barrie, 2019). Robots are also used to increase productivity in warehouses. At Uniqlo’s flagship warehouse, robots have already replaced 90% of its human workers (Nishimura, 2019a). Gap has also adopted a robot system for picking and sorting merchandise, with a plan to triple its deployment of robots to 106 by fall 2020 (Warren, 2020).

Intelligent manufacturing automates not only production but also complex optimization decisions. An example is Adidas’ robot shoe factory, called a “speed factory”, built in Germany and Atlanta in 2016 and 2017, respectively. It digitizes and automates the sneaker production through digital design, computerized knitting, robotic cutting, and 3D printing, shortening the lead time from months to days. Both factories, however, closed in 2019 because the factories failed to achieve the economies of scale and scope to remain profitable. Adidas will instead utilize the technologies in their factories in Asia (Thomasson, 2019). Hyosung TNC, a Korean textile company, has installed intelligent manufacturing systems in its spandex factories in China and Vietnam. The systems collect and analyze data from the entire supply chain, from raw material import to production and shipment. For example, machine vision can identify defective products by analyzing data obtained from a high-speed camera. The real-time monitoring processes result in consistent quality products (Friedman, 2019).

Robotics and intelligent manufacturing enhance productivity by reducing production time and enhancing manufacturing efficiencies (Wellener et al., 2019), and by adapting to changing demands in real-time. Furthermore, the shorter lead time and minimal human intervention allow factories to be located closer to consumers, better positioned to respond to their ever-changing demands with agility, which indirectly helps reduce unsold inventory, thereby enhancing environmental sustainability.

#### 3D printing and 3D knitting

3D printing, also known as additive manufacturing, creates 3D objects from a digital file. It is an additive process whereby an object is created by adding successive layers of materials, referred to as filaments. Each layer is a thinly sliced horizontal cross-section of the final output (Berman, 2012). A print head or extruder melts the filament and turns it into a 3D model. On the other hand, 3D knitting uses needles to produce knit products in one piece, without the need for sewing (Miodownik, 2015). Both technologies enhance efficiency and customizability by reducing manual labor and production processes (Conner et al., 2014; Sun & Zhao, 2017).

3D printing application in the fashion industry has been limited to accessories and, most prominently, footwear, because materials that can be used for 3D printing are mostly plastics, which are unsuitable for garments. The adoption in the fashion industry has primarily been in haute couture as an experiment for innovative designs (Vanderploeg et al., 2017). Examples of 3D printing applications in the footwear market include Nike’s Flyprint sneakers with 3D printed upper, and Adidas’ Futurecraft and Alphaedge sneakers with 3D printed mid-soles. Conventional sneaker manufacturing requires a metal mold to produce soles. While it takes more than a month to build a mold, let alone the sole, 3D printing reduces the production time to less than two hours (Bain, 2017). In addition, the 3D-printed upper can be customized to an individual runner’s foot (Wilson, 2014). Two American start-ups, Feetz Shoes and Prevolve, offer customized 3D-printed shoes (Park, 2020). Feetz allows its customers to use its app to measure their feet size at home by taking three photos of each foot. They can then choose the shoe style and color. Within 7 days, customized 3D-printed shoes are delivered to customers (FDRA, 2020). Prevolve uses a professional foot scanner at its office located in Seattle, WA, to get an accurate 3D model of customers’ feet (Prevolve, n.d.). Prevolve’s customers need to schedule an appointment and visit the physical location for scanning. Wiivv, a Canadian start-up founded in 2014, offers 3D-printed custom-fit sandals, which can be ordered on its app. Its sandals have custom arch support as well as custom strap and toe thong placement (Freudmann, 2020).

Relative to 3D printing, 3D knitting has been more widely adopted by both B2B and B2C fashion companies, as this technology uses conventional yarns for materials. Companies utilizing 3D knitting include B2B companies that produce 3D knit garments, such as sweaters for small and medium companies, such as Tailored Industry based in NY, USA, and 22 factor based in Hong Kong. The technology allows the companies to manufacture in small batches or on demand, drastically reducing excess inventory (Friedman, 2019; Tailored Industry, n.d.). B2C companies utilizing 3D knitting include Ministry of Supply, a Boston-based high-performance business casual brand, and Son of a Tailor, a men’s custom-made casual wear brand based in Denmark. Ministry of Supply offers an option to customize 3D-printed knit jackets in-store. Customers can choose the size, yarn, button, and color (Shima Seiki, n.d.). These brands highlight how the technology reduces material wastes to almost zero, compared to 21–35% of fabrics for traditionally made garments (Ministry of Supply, n.d.; Son of a Tailor, n.d.).

3D printing and 3D knitting technologies are leveraged to enhance productivity and hyper-personalization. They reduce waste, lead time, and production costs by minimizing manual labor and simplifying the production process. The lean operation and digitization make it possible to achieve hyper-personalization, which is costly to scale with conventional means (Paul, 2001).

#### Virtual and augmented reality

Virtual reality (VR) technology creates a computer-generated digital environment, which supplants the user’s real-world environment. The fully artificial environment can replicate a real-world environment or render an imaginary one (Kunkel, 2016). On the other hand, augmented reality (AR) technology superimposes computer-generated content onto the user’s real-world environment, thus blending digital and physical worlds (Milgram et al., 1995). VR and AR technologies have improved immensely since their invention in the late 1950s and 1960s (Javornik, 2016; VRS, n.d.). Improvements in capabilities, coupled with decreasing costs and emerging ecosystems, have led to more widespread adoption and applications across industries (Fitzgerald et al., 2018).

In the fashion industry, both retailers and brands experimented with technologies to enhance in-store and online shopping experiences. These retailers and brands encompass casual, fast fashion, luxury, and contemporary from Gap and Zara to Neiman Marcus and Rebecca Minkoff to name a few. While some deploy in-house capabilities, many retail companies’ VR and AR initiatives are supported by emerging third-party companies specializing in fashion retail.

A VR example is Metail, a London-based virtual fitting room technology company, established in 2008. With Metail’s technology, online shoppers can create their 3D avatars by uploading their photo and body measurements. The avatars reflect the shoppers’ body shape with 92% to 96% accuracy. The shoppers can try on outfits by superimposing products over their avatars and rotating them 360 degrees. A retailer that has utilized this technology is Tesco, which offered a virtual fitting room on its Facebook page in 2012 (O’Hear, 2012). VR application is not limited to consumer use. Metail’s EcoShot, which is an add-on feature within VStitcher, 3D fashion design software, allows designers to simulate garments on 3D renditions of real people. The software enables designers to get a sense of how their designs will look on their target consumers, without having to do collaging or photoshop work (Metail, n.d.).

Gap, Inc. experimented with AR technology by launching DressingRoom mobile application, in partnership with Avemeric, a digital 3D software company founded in 2012. This application allowed a customer to superimpose products on a virtual mannequin customized to the customer’s body measurements, which was projected onto the real-world environment. The goal was to allow customers to see the draping and fitting of garments without having to physically try on products (O’Shea, 2017). Neiman Marcus and Rebecca Minkoff use MemoMi, a smart mirror software company founded in 2013. MemoMi’s interactive mirror that utilizes AR technology allows customers to try on products virtually and view outfits from 360 degrees (Memomi, n.d.).

Additionally, increasingly more brands are incorporating VR and AR into their marketing campaigns (Mcdowell, 2020). For example, Puma’s LQD CELL Origin AR, its augmented reality shoe, allowed users to play interactive games (Puma, 2019). Zara’s AR mobile application allowed shoppers to view AR images of fashion models, such as Fran Summers, wearing their products. The shoppers could view VR images at multiple touchpoints, including in-store podiums and Zara.com (O’Shea, 2018). Besides the experiential value (Watson et al., 2018), VR and AR can offer utilitarian values, such as saving time and effort. For consumers, while current technologies are not yet advanced enough to render accurate fit and draping of varied fabrics or 3D models of the customer’s body, they have the potential to deliver more detailed product information than conventional web experiences (Cook et al., 2020). If the level of realism increases to meet customer expectations, a virtual try-on may completely replace an actual try-on. As a result, consumers may no longer need to physically try on items to find the best fit, thereby significantly reducing the time and effort spent on finding the right products, whether it be visiting a physical store or making multiple online orders/returns. For fashion designers, virtually testing the products with 3D models of target consumers can save time and effort by obviating the need for a physical fitting testing.

These industry examples demonstrate that VR and AR technologies can be utilized to enhance productivity and hyper-personalization. They have the potential to increase efficiency in both consumers’ shopping decisions and designers’ product development processes. The technologies’ flexibility aids hyper-personalization, as shown in the case of custom-sized virtual models of consumers. The next technology the 4th Industrial Revolution brought to us is artificial intelligence.

#### Artificial intelligence

Artificial intelligence (AI) refers to machines that mimic cognitive functions typically associated with humans, such as pattern-recognition, perception, and learning. It is governed by a set of algorithms, which is a set of rules a computer follows to make a prediction or solve a problem by processing and recognizing patterns in a large volume of data (i.e., big data). Unlike humans, AI can handle big data reliably without fatigue. Therefore, the technology makes extracting insights from big data less cumbersome, enhancing scalability (Koehler, 2018). Increasingly, AI has been driving corporate decisions and customer values, from setting prices to recommending products (Iansiti & Lakhani, 2020).

AI-enabled predictions are also informing or automating a range of corporate decisions in the fashion industry from marketing, pricing, and inventory management to product development. By analyzing real-time retail data on competitors’ products, pricing, and promotional communications, brands can make informed product, marketing, and pricing decisions. AI can also help analyze consumers’ interests, buying trends, and automatically adjust inventory, based on real-time demand (Edited, n.d.). Based on detailed garment specifications and style attributes, AI can recommend the right sizes to customers. It can also recommend items tailored to individual preferences, based on previous purchase history, customer preferences, and feedback. Furthermore, AI can analyze the attributes of best-selling items and create new designs with the highest possibility of becoming the next bestsellers.

These AI capabilities are either built in-house or outsourced to B2B service providers (Edited, n.d.). A B2B example is True Fit, a Boston-based company founded in 2005. It offers hyper-personalization services powered by AI, such as online product catalog personalization and size recommendation services. Based on retail big data, including millions of detailed product specifications and attributes, sales data, and customer profiles, the company allows brands to personalize the product page of their online stores and help online shoppers find the correct sizes. To create seamless experiences, its services are integrated into the brands’/retailers’ websites. True Fit’s clients include 28 retailers and brands such as Levi’s, Macy’s, Ralph Lauren, Aldo, and Kate Spade (Kapner, 2019).

A prime B2C example is Stitch Fix, an online styling service company founded in 2011. By integrating and analyzing customer data from multiple sources, Stitch Fix curates and delivers five items tailored to an individual customer. The curated items comprise products of existing brands as well as in-house labels. In addition to the styling service, algorithms guide all types of business decisions, including logistics, inventory management, and product design. In designing products, to maximize the odds of creating a bestseller, Stitch Fix uses algorithms to analyze sales data and identify popular product attributes. Then, they are combined to create a new style (Hernandez, 2017). Another B2C example is Anomali, a custom wedding dress brand founded in 2016, headquartered in San Francisco. Using its AI-powered DressBuilder survey, customers can customize different elements of a wedding dress, such as silhouette, lace, neckline, and train to create a one-of-a-kind wedding dress. With 4 billion dress options, the customization possibility is virtually limitless (Nishimura, 2019b).

In sum, by analyzing a large volume of both historic and real-time data on consumers and products, AI enables firms to develop and find personalized items for individual customers. In addition, it helps fashion companies make more effective and accurate decisions in important areas, such as marketing and pricing, and substantially lower the operating cost while improving the accuracy of demand forecasts.

### Meeting the prime goals without the 4IR technologies

This section reviews start-up cases that address one or more of the three prime goals without the 4IR technologies. Start-up companies satisfying hyper-personalization needs include Indochino. Vancouver-based Indochino, an online custom menswear brand, allows customers to shop for custom-made suits, shirts, casual pants, and blazers in the comfort of their home. The company offers a wide range of customization options. For jackets alone, Indochino offers 14 options, including lapels and linings (Federico-O’Murchu, 2015). While Indochino uses algorithms to detect errors in customers’ self-reported measurements, their business primarily relies on user-friendly online customization interfaces, instructional videos for taking measurements, and showrooms where customers can receive customization and styling services in person (Hemmadi, 2016). In 2019, Indochino was ranked the third fastest growing brand in Canada for businesses with revenues of more than $100 million and the first as the fastest growing Canadian retailer internationally (Cision, 2019).

Start-up cases that successfully address environmental sustainability needs include Rent the Runway, an online fashion rental company, and The Real Real, an online luxury consignment company. These two cases represent collaborative consumption, a consumption mode characterized by sharing activities, such as renting, reselling, and trading of goods (Belk, 2014). Rent the Runway offers a rental service that allows customers to consume a variety of fashion items at a fraction of the price for a short period. The Real Real sells pre-owned luxury items on a consignment basis. The company allows sellers to monetize their underused items and buyers to acquire luxury products at substantially lower prices. With renting and exchange platforms for second-hand goods, respectively, each offers consumers more sustainable options with much lower budgetary constraints. Rent the Runway’s rental model and The Real Real’s reselling model maximize the product life cycle through reuse and encourage consumers to buy less. They promote a circular economy, where resource usage and waste are minimized (Geissdoerfer et al., 2017). Without utilizing any 4IR technologies, in the first quarter of 2020, the company’s gross profit increased 16% year over year, $49.2 million, despite the overall decrease in retail spending amidst Covid-19 (The Real Real, 2020). Now valued at $1B, Rent the Runway raised an additional $125 million in its latest fund-raising round in 2019, totaling $337 million (Maheshwari, 2019).

Of start-up examples addressing productivity needs without utilizing core 4IR technologies, JOOR is a good case. Founded in 2010, JOOR is an online wholesale platform that enhances productivity through the digitization of the wholesaling process (Joor, n.d.). Traditionally, wholesaling requires attendance at various trade shows. Instead, JOOR creates a digital catalog for the buyers to sort through with advanced search tools and place orders online, increasing efficiency for both brands and retailers. For brands, the company also provides real-time data on incoming orders and inventory. As a result, the entire process from an appointment to orders only takes 45 min; 45% faster than the traditional method (Joor, n.d.). The company now connects over 8600 brands with 200,000 retailers across 144 countries (Joor, n.d.). In 2019, Joor raised $16 million in a Series C funding round, totaling $36 million (Singh, 2019).

The above examples demonstrate a clear message: fashion start-ups can address the three prime goals without utilizing 4IR core technologies. This attests that the technologies are enablers, not the essence on which a fashion company is built. The key to their success lies in innovations in their business model (Zott et al., 2011), not in their technologies. A business model specifies how the firm creates value (i.e., value proposition) and how it captures value (operation model). A value proposition specifically addresses what and for whom a firm is offering, while the operating model deals with how the offering can be delivered profitably (Jin & Shin, 2020). The aforementioned cases (Indochino, Rent the Runway, the Real Real, and Joor) clearly specify their unique value propositions and operating models to deliver new value propositions not emphasized by incumbents. Rent the Runway’s key value proposition is offering a rental service, thereby offering consumers more choice and extending the product lifecycle (thus being sustainable). The point is that it was not the utilization of advanced technology, such as AI, but the company’s thoughtful and innovative business model that created value for customers. The primary lesson is that the 4th Industrial Revolution technologies are not a precondition to the successful delivery of the prized consumer values. While Rent the Runway uses algorithms to support business decisions, such as buying decisions, as well as complementary services, such as a product recommendation service, what drives their main business is a traditional logistics system, albeit a complex one, rather than advanced technologies (Scott, 2020). Similarly, The Real Real’s business is largely supported by a conventional e-commerce logistics system (The Real Real, n.d.). Table 1 summarizes which prime goal each case had addressed. Productivity is embedded into their business models, not necessarily the goals, except robotics and intelligent manufacturing. It will then be important to understand how each of the three prime goals was addressed.

**Table 1:** Three prime goals addressed by the examples

Technology	Examples	Prime Goals		
		Personalization	Environmental sustainability	Productivity
Robotics/intelligent manufacturing	Sewing robots			$\downarrow$ Production time $\uparrow$ Efficiency
	Warehouse robots			
	Smart factory		✓	
3D Printing/3D knitting	Adidas	✓		$\downarrow$ Production time $\uparrow$ Efficiency
	Nike	✓		
	Tailored Industry		✓	
	Ministry of Supply	✓	✓	
	Son of a Tailor	✓	✓	
	22 Factor	✓	✓	
Virtual/augmented reality	Gap’s Dressing Room	✓		$\downarrow$ Decision-making time $\uparrow$ Convenience
	Metail	✓	✓	
	EchoShot by Metail	✓		
	MemoMi	✓		
	Feetz	✓		
	Prevolve	✓		
Big data analytics/ AI	Stitch fix	✓		$\downarrow$ Decision-making time $\uparrow$ Accuracy and Convenience
	True fit	✓	✓	
	Anomali	✓		
Business model innovation	Indochino	✓		$\downarrow$ Decision-making time $\uparrow$ Convenience
	Rent the runway		✓	
	The RealReal		✓	
	Joor		✓	

## Discussion

This study delineated that the 4th Industrial Revolution is evolving to satisfy the three prime goals in the fashion industry: hyper-personalization, environmental sustainability, and productivity. Below, we integrate *how* companies in this study address the three prime goals with either technologies or innovative business models.

The first prime goal for hyper-personalization is met in multiple ways. Here, ‘personalization’ goes beyond making mass-customized clothes for consumers, which was made possible during the 3rd Industrial Revolution. The 4th Industrial Revolution technologies are aiding fashion brands by lowering the cost of offering personalized products and services (Wang et al., 2017). Using 3D printing technology, 22 factor, Ministry of Supply, Wiiv, Feetz Shoes, and Prevolve produce personalized products more extensively. The AR and VR technologies can enable personalized services, such as a virtual try-on with a 3D rendition of online shoppers that reflects the individual consumer’s body shape with great accuracy (e.g., Metail and Gap’s DressingRoom). Likewise, AI helps provide services and products tailored to an individual customer, whether it be finding the right fashion items (e.g., Stitch Fix, designing a one-of-a-kind wedding dress, e.g., Anomali), or the right sizes (e.g., True Fit).

The next prime goal, environmental sustainability, is also met with or without 4IR technologies. Intelligent manufacturing can optimize not only energy use but also the production process, leading to fewer defects and recalls, thus less waste (Wang et al., 2016). 3D printing and 3D knitting technologies enable made-to-order production without leftover scraps, reducing production waste toward zero. The virtual try-on feature supported by VR and AR technologies can mitigate the primary risk of shopping online, reducing the likelihood of returns. Similarly, AI-powered services that help customers find the right products can reduce returns, resulting in a lower carbon footprint (True Fit, n.d.). Without 4IR technologies, Rent the Runway, The Real Real and Joor address concerns for environmental sustainability by re-use (e.g., Rent the Runway), extending the product lifecycle (e.g., The Real Real), and reducing travel to wholesale shows (e.g., Joor).

The third prime goal, productivity, is manifested in many aspects; the concept of productivity is broadened to include not only tangibles such as the volume of material goods, but also intangibles such as accuracy, efficiency, and convenience in decision-making—both companies and consumers—as the output. Advanced robotics is beginning to automate sewing, the most labor-intensive task in apparel production. Intelligent manufacturing, which automates decision-making through self-optimization, self-diagnosis, and problem-solving, is expected to triple the productivity growth rate during the next decade (2019–2030) (Wellener et al., 2019). 3D printing and 3D knitting technologies eliminate the need for sewing and completely automate the production. Companies such as Metail and MemoMi utilize VR and AR technologies to help consumers save decision-making time and effort by allowing them to try on items virtually. Similarly, with AI, companies like Stitch Fix, True Fit, and Anomali save consumers’ decision-making time and effort, by helping consumers to find their right styles and sizes, so further enhancing convenience and efficiency. AI likewise improves the productivity of companies by automating the delivery of their services. Here, it should be noted that four successful start-up cases without 4IR also save consumers decision-making time and enhance convenience through their innovative business models.

Table 2 summarized how each start-up case addresses the three prime goals. In summary, the need for hyper-personalization is met by offering personalized production, a variety of choices, or personalized experience through helping consumers find their right choices, such as True Fit and Stitch Fix. The need for environmental sustainability is addressed by reducing material waste and unsold inventory, re-using the product and thereby extending the product lifecycle, using rental services, or saving travel time to wholesale shows or product launch meetings. Productivity is met through automation in production as well as decision-making, enhancing accuracy and convenience, not necessarily enhancing production volumes.

**Table 2:** How 4IR addresses the three prime goals + Future research agenda

Prime goals	How 4IR addresses the prime goals	Future research agenda
Hyper-personalization	Personalized products/services More variety at a reduced cost to satisfy diverse needs Assist consumers in finding the right choices	Enablers, facilitators and inhibitors of consumers’ use of personalized products/services Enablers, facilitators and inhibitors of fashion companies’ offering of personalized products/services Consumer motivation and satisfaction toward the utilization of diverse 4IR technologies Profiling of early adaptors of personalized products/services
Environmental sustainability	Reduce material waste and unsold inventory Re-use and extend product lifecycle through a rental service Save travel time	Consumers’ willingness to adopt 4IR technologies that boost sustainability Enablers, facilitators and inhibitors of fashion companies’ utilization of 4IR technologies that solve sustainability concerns Fashion companies’ motivation and willingness to develop innovative business model that solve sustainability issues Descriptive analyses on consumers as well as companies’ sustainable practice
Productivity	Automation in production as well as decision-making Increase accuracy, efficiency, and convenience, not necessarily production volume	Performance implications of company adaption of 4IR technologies Assessing the accuracy of assisting consumers in finding the right choices Fashion companies’ challenges in utilizing 4IR technologies Factors related to companies’ willingness to invest in 4IR technologies that enhance productivity Consumer evaluation of 4IR technologies that offer convenience and assist their optimal decision making

## Conclusions

Powered by 4IR technologies and innovative business models, the fashion industry has brought back the personalized production consumers enjoyed during the craft production era prior to the 1st Industrial Revolution (Koren, 2010). From the 1st to 3rd Industrial Revolution, enhancing productivity was the main goal, which was met through mechanization (1st Industrial Revolution), mass production (2nd Industrial Revolution), and digitalization and automatic production (3rd Industrial Revolution). Through the 1st to 3rd Industrial Revolution, consumers had plentiful product options, but they were made to satisfy the preferences and needs of the average consumer and to appeal to a larger audience. Despite the sufficient volume that democratized consumption, both companies and consumers faced a tradeoff owing to unmatched demands: consumers often had to settle with “good enough” products, and companies were routinely left with excess inventory. At the same time, unmatched demand resulting from mass production and leftover inventory often created waste and landfill harmful to the environment. In this era, consumers now desire fashion products with styles and fit reflecting their color and design preferences rather than mass-produced items made for the average consumer. A prime example of a fashion brand attempting to meet this need via 4IR may be Stitch Fix, which provides a personalized styling service utilizing AI and big data.

4IR will effectively handle the major concerns of the industry pointed out in this study: unmatched demand and oversupply. It occurs because more products were produced than demand without accurately knowing consumers’ needs. With personalized production and service, there will be less inventory, so the world will become more sustainable. Satisfaction levels for both consumers and firms will be heightened: Consumers can find the right design, price, and fit for them, and firms will have less unsold inventories, all of which contribute to environmental sustainability. The two prime goals, therefore, are interrelated: one enhancement (e.g., hyper-personalization) contributes to the enhancement of another (e.g., environmental sustainability). The 4IR technologies and innovative business models are enablers that aid the effective achievement of the goals of hyper-personalization and environmental sustainability with heightened accuracy, convenience, and ease of decision-making.

### Managerial implications

With the changes that COVID-19 brings, the speed of 4IR will be accelerated, and the impact will be more pervasive. The rise of a contact-free economy has prompted many consumers to shop online and pushed fashion firms to conduct business digitally. As such, digitizing the end-to-end supply chain will require investments in innovative technologies. However, as integrated into the above section, the three prime goals can be successfully met with or without 4IR technologies. 4IR technologies are critical enablers that help meet the needs in a scalable way. When technologies are first introduced, they are often treated like a panacea emphasizing the merits. When dot.com emerged in the early 2000s, the ability to develop and operate a website was considered a huge skill. It did not take much time for firms to learn that merchandising skills that satisfy consumers’ needs are more critical than developing and operating e-commerce sites. At present, many tools and apps needed for website development and operation are widely available to the level that individuals can easily develop their own websites. The global fashion industry should learn from this history. That is, once 4IR technologies are widely available, companies with innovative business models that address consumers’ unmet needs will flourish, and 4IR technologies will serve as tools. Therefore, fashion companies should prioritize understanding constantly changing consumer needs to inform their decisions on business model innovations. It is because profiting from technology or product innovation still requires an innovative business model (Teixeira, 2019). Technologies cannot be an end goal and are enablers for a positive change. They have been deployed to advance human life (Schwab, 2016). It is business model innovations that propel fashion firms to move forward. That is why resource-deficient small start-ups can outperform the incumbents without leveraging 4IR technologies.

### Future research agenda

The three prime goals proposed and further discussed with company cases provide important implications for research, summarized in Table 2. Among the three prime goals addressed in this study, the most influential goal of the fashion business may center on hyper-personalization because it will bring fundamental changes in marketing and branding. If hyper-personalization becomes a norm in the fashion industry, the traditional marketing and branding approaches developed with the assumption of mass consumption will have limited effectiveness. Therefore, new marketing and branding tactics will need to be invented to address hyper-personalized consumption in all 4p’s: product development, pricing, place of distribution, and promotion method. This indicates that research should be directed to identify the effectiveness of new marketing and branding strategies, including consumers’ responses and perception toward new approaches.

Second, this study identified how the 4IR technologies and innovative business models address the three prime goals. In each approach summarized in Table 2, research can be directed to understand the enablers, facilitators, and inhibitors of consumers’ willingness to adopt a new approach in each of three prime goals (i.e., hyper-personalization, environmental sustainability, and productivity). For example, what are the factors that inhibit consumers from the use of personalized products made possible with 4IR technologies such as 3D printing or 3D knitting? What is consumer perception of and attitude toward products designed by AI? Would consumers perceive products designed by AI as uncreative or inspirational? What is the optimal role of AI in the consumer’s mind? Would consumers not care who made the products as long as the products accurately meet their preferences? In addition, studies can be directed to understand consumers’ motivation and satisfaction toward products recommended by AI and to compare consumer satisfaction among various AR- and VR-based services that assist consumers in finding the right products. Therefore, a study on profiling regular users of the personalized service will provide fashion companies with practical insights.

While prior research substantially contributed to the conceptual understanding of 4IR technologies’ applications (e.g., Braglia et al., 2020), research examining them from the consumer perspective is limited to certain applications (e.g., AI-enabled service vs. product), certain technologies (e.g., AI and VR/AR), and noncomparative research (e.g., single type of AR-based virtual try-on service; Lee et al., 2021). Investigating under-researched technology (e.g., 3D knitting) and applications (e.g., AI-designed products) as well as conducting more comparative research (e.g., comparing VR and AR services) will offer further insights that can inform future directions.

In addition, it would be useful to examine potential conflicts between hyper-personalization and environmental sustainability. An unintended consequence can be lower perceived resale value as a result of products made to fit unique body measurements of an individual consumer. As such, identifying and examining potential conflicts would offer a more nuanced understanding of the intersection of hyper-personalization and environmental sustainability.

Third, for fashion companies, the utilization of 4IR technologies requires substantial investment and training. Developing a new business model within an incumbent fashion company can be a huge challenge as it is likely to be incompatible with the existing operating structure. Some companies may be nimble enough to respond timely to these changes, while others may not be able to overcome the internal inhibitors. Therefore, identifying organizational barriers and drivers of the adoption of 4IR technologies and innovative business models, in addition to how companies can align the technologies with their business models will help address this knowledge gap.

Despite growing research interest on this topic (e.g., Horváth & Szabó, 2019), prior research focusing on the fashion industry is very limited, even for one of the most researched types of 4IR technology (i.e., big data analytics; Mariani & Wamba, 2020). In addition, investigating how fashion start-ups identify and exploit opportunities for business model innovations that address the aforementioned prime goals will help develop a unified framework for the entrepreneurial thinking process. The current literature on the intersection of business model innovation and entrepreneurship in the fashion industry is limited to answering the question of what startup brands’ business model innovations are. Thus, more research is needed to uncover the process (i.e., “how”) of developing business model innovation (Jin & Shin, 2020; Todeschini et al., 2017).

Fourth, a stream of research can be directed to assess the productivity that the 4IR technologies and innovative business models can bring. In other words, an understanding of how the proposed 4IR technologies can translate into the performance of a fashion company will provide useful implications for development. In addition, as the 4IR technologies are proposed to help meet the three prime goals, future studies can verify whether they really enhance the productivity broadly defined in this study. They can also investigate whether consumers can perceive styles or designs recommended by AI as accurate and can thus enhance their decision-making effectiveness.

Finally, but not least, this study proposes several questions that the industry and academia alike need to consider: If personalized products and services are the key, is the current branding approach still relevant? How can fashion companies market their goods effectively? If AI can design, how will the role of fashion designers change? If AI can select bestselling items, how will this affect buyers’ responsibilities? Should current academic curriculum be revised based on these changes? Personalized production does not require volume production; then, the popularity of global sourcing will wane and personalized production closer to consumers—such as reshoring or onshoring—will gain momentum. The reshoring idea is closely related to the environmental sustainability concept as it will reduce unsold inventory via shortened lead times and more frequent and smaller orders, which ultimately reduces the environmental impact (Budding & Martin, 2021). How can fashion companies make reshoring or onshoring possible? What are the enablers, inhibitors and facilitators of onshoring? If global sourcing becomes less popular, how can a country effectively produce onshore? Can this create jobs in a country where labor cost is high? With automation, robots, and intelligent manufacturing, can fashion goods be produced in economically advanced countries?

### Limitations and future studies

In this study, the three prime goals are proposed based on two major challenges in the fashion industry: unmatched demand and oversupply. The analyses on sustainability, accordingly, is mainly from the environmental perspective because it is a direct consequence of unsold inventory owing from unmatched demand and oversupply. According to the triple bottom view (or perspective), three pillars of sustainability (i.e., social, economic and environment) should be well balanced (Elkington, 1998). For future studies, therefore, social and economic aspects of sustainability should be further analyzed with cases. The insights gained from this study are mainly from successful cases in Western countries. Future studies may substantiate the current study’s conclusions by conducting qualitative and quantitative studies with the industry as well as consumers. While less documented, there might be innovative cases in Eastern and emerging countries, which warrant further examination for a richer, more comprehensive understanding.

## Data Availability

Not applicable.
